# Evaluation of Zosteric Acid for Mitigating Biofilm Formation of *Pseudomonas putida* Isolated from a Membrane Bioreactor System

**DOI:** 10.3390/ijms15069497

**Published:** 2014-05-28

**Authors:** Andrea Polo, Paola Foladori, Benedetta Ponti, Roberta Bettinetti, Michela Gambino, Federica Villa, Francesca Cappitelli

**Affiliations:** 1 Dipartimento di Scienze per gli Alimenti, la Nutrizione e l’Ambiente, Università degli Studi di Milano, via Celoria 2, 20133 Milano, Italy; E-Mails: andrea.polo@unimi.it (A.P.); michela.gambino@unimi.it (M.G.); federica.villa@unimi.it (F.V.); 2 Department of Civil and Environmental Engineering, University of Trento, via Mesiano 77, 38123 Trento, Italy; E-Mail: paola.foladori@ing.unitn.it; 3 Dipartimento di Scienze Teoriche e Applicate, Università degli Studi dell’Insubria, via Valleggio 11, 22100 Como, Italy; E-Mails: benedetta.ponti84@gmail.com (B.P.); roberta.bettinetti@uninsubria.it (R.B.)

**Keywords:** antifouling system, biofilm, *Pseudomonas putida*, wastewater treatment plant, membrane bioreactor, ecotoxicological tests

## Abstract

This study provides data to define an efficient biocide-free strategy based on zosteric acid to counteract biofilm formation on the membranes of submerged bioreactor system plants. *16S* rRNA gene phylogenetic analysis showed that gammaproteobacteria was the prevalent taxa on fouled membranes of an Italian wastewater plant. *Pseudomonas* was the prevalent genus among the cultivable membrane-fouler bacteria and *Pseudomonas putida* was selected as the target microorganism to test the efficacy of the antifoulant. Zosteric acid was not a source of carbon and energy for *P. putida* cells and, at 200 mg/L, it caused a reduction of bacterial coverage by 80%. Biofilm experiments confirmed the compound caused a significant decrease in biomass (−97%) and thickness (−50%), and it induced a migration activity of the peritrichous flagellated *P. putida* over the polycarbonate surface not amenable to a biofilm phenotype. The low octanol-water partitioning coefficient and the high water solubility suggested a low bioaccumulation potential and the water compartment as its main environmental recipient and capacitor. Preliminary ecotoxicological tests did not highlight direct toxicity effects toward *Daphnia magna*. For green algae *Pseudokirchneriella subcapitata* an effect was observed at concentrations above 100 mg/L with a significant growth of protozoa that may be connected to a concurrent algal growth inhibition.

## 1. Introduction

Membrane-based separation processes have gained increasing interest over the last decade, and are becoming the promising technology in wastewater treatment as well as in drinking water and high purity water production and purification in biorefining and bioenergy processes [1,2,3,4].

Membrane technology is attractive to complement or supplant conventional filtration and sedimentation processes and it is also now being integrated into membrane bioreactor systems (MBR) as a reliable and advanced option to improve the performances of the conventional activated sludge processes in wastewater treatment plants (WWTPs) [5]. Membrane based separation processes often offer the following significant advantages over conventional unit operations: easy scale-up and flexibility due to modular design, production of a very high quality effluent, simplification of operation and great reduction in space requirements [6]. In contrast, a major disadvantage is high energy consumption due to the significant aeration intensity applied in MBR for biofouling control. Indeed, the rapid decline of the permeate flux over time due to biofouling is recognized as the major obstacle in the application of membrane technologies [7].

Biofouling is an operational definition, referring to the undesirable accumulation of microorganisms in form of biofilm on membrane surface [8]. Biofouling increases feed flow pressure, extending system downtime, operation costs and decreasing the lifespan of the membrane modules and process productivity [9].

Antifouling strategies for membrane-based separation processes have focused mainly on the optimization of operating parameters [10,11], membrane surface modification [12], and chemico-physical cleanings [13]. However, the influence of variations in the operation parameters on microbial communities is still scarce and in full scale WWTP it is not possible to control oscillations in the composition of the wastewater or environmental variables [11]. The membrane surface modifications have also several disadvantages: mainly the flux declines rapidly and thus frequent backwashing is necessary, the effluent quality is unstable especially after regeneration, the main module configuration applied is flat sheets for which the packing density is low and non-renewable materials are often used [12]. Physical cleaning and antimicrobial agents are not effective in controlling biofouling since biomass is not effectively removed from surfaces, resulting in rapid bacterial regrowth and might provide nutrients for subsequent colonization [14,15,16]. Indeed, some biocides increase the nutrient content by oxidizing recalcitrant organics, making them more bioavailable and improving microbial growth [8]. In addition, it is well established sessile microorganisms express phenotypic traits that are distinct from those that are expressed during planktonic growth, displaying a much higher tolerance to antimicrobial agents [17]. Some mechanisms that may protect biofilms are limited agent penetration, presence of stationary phase dormant zones and existence of a subpopulation of resistant phenotype in the biofilm [18]. After exposure to the killing effects of biocides, a small surviving population of persistent bacteria can rapidly repopulate the surface, and become more resistant to further biocide treatment [19]. Finally, the growth in public concern over the use of biocides may pose a regulatory risk for applications in water treatment.

To guarantee minimal biofilm coverage on membrane surface for extended time, a novel biocide-free approach aimed at interfering with the key steps of biofilm genesis is proposed. *P*-(sulphooxy) cinnamic acid sodium salt (zosteric acid) represents an antifouling agent able to significantly reduce, at sublethal concentrations, both bacterial and fungal adhesion and to successfully counteract the effects of colonization-promoting factors like temperature and time [20,21]. Therefore, a strategy based on zosteric acid could extend the lifespan of the membrane modules and process productivity preventing the formation of deeper and stronger biofilms. Biofilms cause an increase in filtration resistance and in transmembrane pressure drop, resulting in increased crossflow velocity—which further forms stronger biofilms—and in a loss of performances in filtration systems [22].

The application of zosteric acid in mitigating biofilm formation on MBRs envisages the incorporation of this promising molecule into a functioning system to resist biofilm formation over a working timescale. Zosteric acid might be immobilized on the surface of engineering systems creating an innovative non-leaching, long-lasting biocide-free material with antibiofilm properties. However, the lack of knowledge on the effects of zosteric acid on the main membrane colonizers and its environmental fate and ecotoxicological impacts should be understood to evaluate development of the new technology.

In this context, the present study aimed to fill these gaps by offering a preliminary study to explore the potential of zosteric acid in mitigating, at sublethal concentrations, biofilm formation by using the target system *Pseudomonas putida* isolated from the membranes of a submerged MBR plant. In addition, for the first time, preliminary screenings of the potential environmental partitioning behavior and ecotoxicological tests of this promising molecule toward the target aquatic organisms *Daphnia magna* and *Pseudokirchneriella subcapitata* were performed. The work is a preparatory study to transfer the compound performance to the membrane-based separation process technology in WWTPs.

## 2. Results

### 2.1. Confocal Laser Scanning Microscopy (CLSM) Analyses of Fouled Filtration Membranes

**Figure 1:**
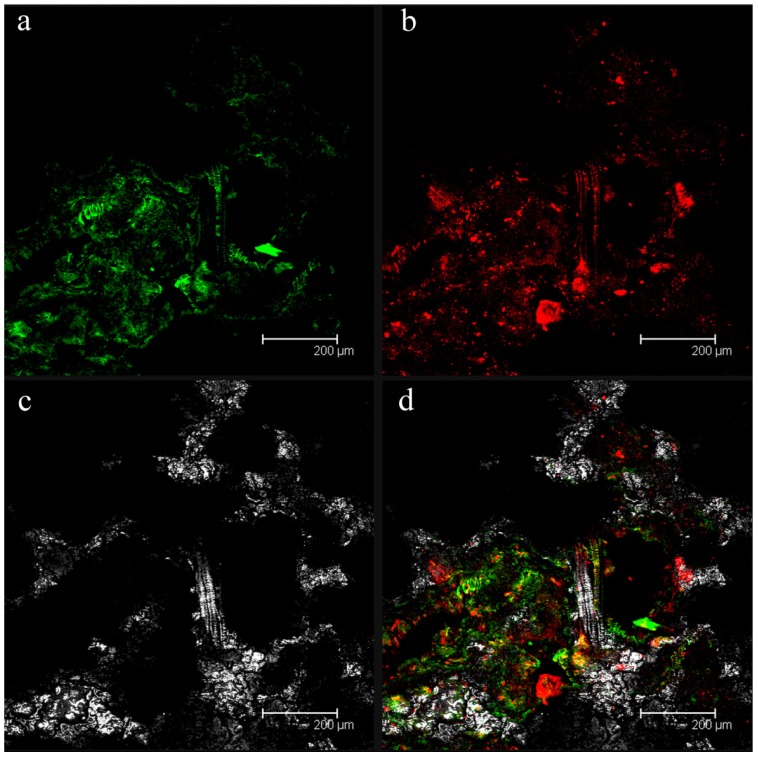
Confocal analysis of the biofouling on the filtration membrane of the Aldeno membrane bioreactor (MBR) wastewater treatment plants (WWTP). (**a**) bacterial cells (green fluorescence); (**b**) the biofilm exopolysaccharides in the matrix (red fluorescence); (**c**) the membrane fibers in reflection mode; (**d**) the superimposition of panels (**a**), (**b**) and (**c**).

Figure 1 illustrates a fouled filtration membrane sample visualized by confocal laser scanning microscopy (CLSM). Microscopic observations showed the presence of sessile cells (green fluorescence) embedded in exopolysaccharides (a component of the biofilm matrix, red fluorescence), thus demonstrating the presence of microbial biofilm.

### 2.2. Denaturing Gradient Gel Electrophoresis (DGGE), Sequencing and Community Profiles Analysis

An accurate understanding of the microbial community composition in membrane systems is crucial to identify target microorganisms. Total DNA was extracted directly from microbial cells collected from sludge and from filtration membrane samples to characterize the fouling community. Table 1 reports the identified strains obtained from denaturing gradient gel electrophoresis (DGGE) analysis. The sequences from sludge and fouled membranes were phylogenetically most closely related to alphaproteobacteria (64%), bacteroidetes (27%) and betaproteobacteria (9%), and to gammaproteobacteria (45%), betaproteobacteria (22%), acidobacteria (11%), bacteroidetes (11%) and other unclassified bacteria (11%), respectively.

**Figure 2:**
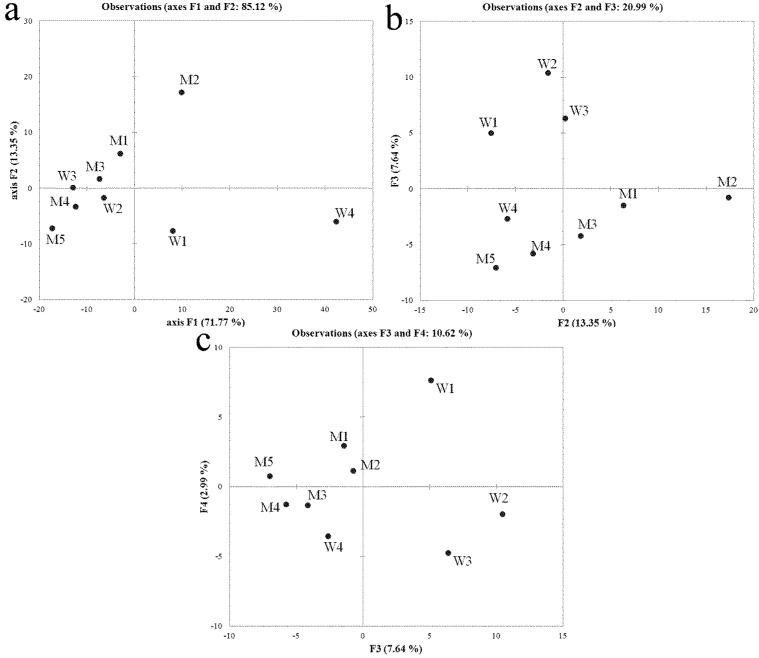
Principal component analysis (PCA-analysis) results for bacterial denaturing gradient gel electrophoresis (DGGE) band patterns. (**a**) First principal component (F1) *vs.* second principal component (F2); (**b**) F2 *vs.* third principal component (F3); (**c**) F3 *vs.* fourth principal component (F4). W, sludge samples; M, fouled filtration membranes.

Principal component analysis (PCA-analysis) was applied to study possible structural similarities in the bacterial communities evidenced by the 16S rDNA DGGE profiles. The plot of the two-dimensional scores accounted for 85.12% of the input data variability for bacteria and was therefore adequate to describe the system under investigation (Figure 2a). The samples W4 and M2 dominated the first principal component (F1, explaining the greatest percentage of the system variability), masking any other interesting grouping among samples. The study of the following principal components (F2 *vs.* F3 and F3 *vs.* F4) was used to gain useful information concerning the differences between sludge samples and samples collected on the membrane (Figure 2b,c). The PCA-analysis excluded a close relationship among bacterial communities as several samples from both fouled membranes and sludge are in separate clusters.

**Table 1:** Identification of partial 16S gene sequences from excised bands observed in denaturing gradient gel electrophoresis (DGGE) profiles.

Source	BlastN reference strains			RDP tassonomic classifier	

	Closest relative strain	Similarity (%)	GenBank accession number	Most probabile taxon	Similarity (%)
Sludge	*Paracoccus* sp.	92	AY646160	Alphaproteobacteria	99
	*Novosphingobium* sp.	99	EU430056	Alphaproteobacteria	100
	*Novosphingobium aromaticivorans*	93	JF459981	Alphaproteobacteria	100
	*Novosphingobium* sp.	96	FM886872	Alphaproteobacteria	100
	Alpha proteobacterium	91	AB578881	Alphaproteobacteria	100
	*Sphingomonas* sp.	99	AJ617690	Alphaproteobacteria	100
	*Sphingomonas* sp.	89	GQ181132	Alphaproteobacteria	97
	Sphingobacteriaceae bacterium	97	FJ386545	Bacteroidetes	100
	uncultured *Niastella* sp.	98	GU326307	Bacteroidetes	100
	Flavobacteriales bacterium	95	EF636194	Bacteroidetes	100
	uncultured Oxalobacteraceae bacterium	91	HM141157	Betaproteobacteria	100

Fouled membranes	uncultured Xanthomonadalesbacterium	89	EF073609	Gammaproteobacteria	99
	uncultured Gammaproteobacteria bacterium	99	CU926678	Gammaproteobacteria	100
	*Rhodanobacter* sp.	99	EU876661	Gammaproteobacteria	100
	*Rhodanobacter* sp.	97	EU876661	Gammaproteobacteria	100
	uncultured Betaproteobacterium	97	JF703550	Betaproteobacteria	100
	uncultured *Comamonas* sp.	89	JF808874	Betaproteobacteria	99
	uncultured bacterium	98	AB241559	Bacteroidetes	100
	uncultured Acidobacteria bacterium	98	HM062044	Acidobacteria	100
	uncultured Betaproteobacterium	89	FJ184029	unclassified bacteria	98

### 2.3. Bacterial Strains Isolated from Fouled Filtration Membrane

Fouler strains were isolated and identified by culture-mediated molecular methods. Table 2 reports the identified cultivable bacterial strains obtained from fouled membranes. The sequences of isolated strains were phylogenetically most closely related to gammaproteobacteria (55%), firmicutes (27%), actinobacteria (9%) and betaproteobacteria (9%).

**Table 2:** Identification of partial *16S* gene sequences from bacterial strains isolated from fouled filtration membrane. PCA, plate count agar medium; SWA, agarized synthetic wastewater medium.

Closest relative strain number	Cultural media	Taxa	Similarity (%)	GenBank accession
*Pseudomonas putida*	PCA	Gammaproteobacteria	99%	JF703662
*Pseudomonas* sp.	PCA	Gammaproteobacteria	99%	HQ718413
*Pseudomonas* sp.	SWA	Gammaproteobacteria	97%	AM689975
*Pseudomonas* sp.	SWA	Gammaproteobacteria	99%	HQ588845
*Shewanella xiamenensis*	PCA	Gammaproteobacteria	99%	HQ418493
*Enterobacter cloacae*	PCA	Gammaproteobacteria	99%	HQ231214
*Bacillus arvi*	PCA	Firmicutes	99%	AM260979.1
*Lysinibacillus fusiformis*	PCA	Firmicutes	99%	GQ844965
*Bacillus cereus*	PCA	Firmicutes	100%	HQ333012
*Rhodococcus* sp.	PCA	Actinobacteria	96%	EU293153
*Chitinimonas* sp.	SWA	Betaproteobacteria	97%	GQ354569

### 2.4. Zosteric Acid Was not a Carbon and Energy Source for the Membrane Colonizer P. putida but Affected Cell Adhesion

The membrane colonizer *P. putida* (accession number HF546530) was used as target microorganism for all the experiments with zosteric acid as it was commonly present throughout the investigated environment and readily forms biofilms [23,24]. In addition, it has a rapid reproduction rate, it was diffusely isolated from activated sludge in WWTPs [25,26], and it has been already adopted as model biofoulant to test antifouling technology on membranes [27,28,29].

Although *P. putida* grew in synthetic wastewater broth (SWB) supplemented with glucose, it did not exhibit growth on zosteric acid as the sole carbon and energy source.

**Figure 3:**
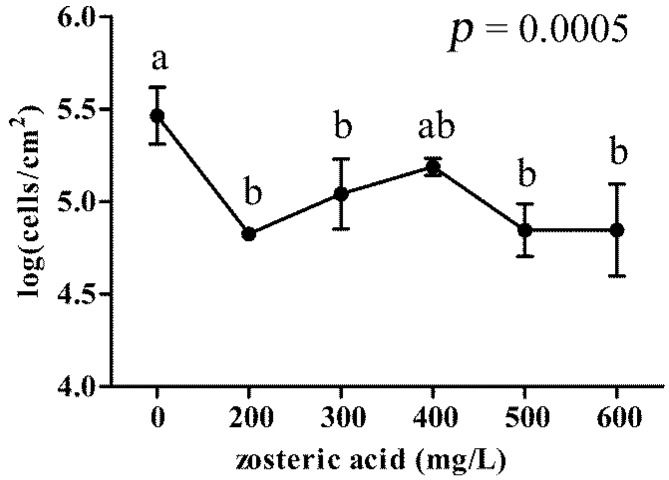
*P. putida* cells adhesion in presence of different concentrations of zosteric acid. In the graph, vertical axis shows log(CFU/cm^2^) values and horizon axis represents different tested concentrations. Each value corresponds to the mean of four replicates. The graph provides the *p*-value obtained by ANOVA analysis. According to post hoc analysis (Tukey–Kramer, *p* < 0.05), means sharing the same letter are not significantly different from each other. Bar errors show standard deviations.

*P. putida* cells adhesion was assessed quantitatively using fluorochrome-labeled cells in hydrophobic 96-well blacksided plates. Bacterial cells stained with 4′,6-diamidino-2-phenylindole dye (DAPI) appeared uniformly labeled and revealed a linear relationship between cell number and fluorescent intensity in a range from 10^2^ to 10^7^ cells. Figure 3 shows *P. putida* cells adhered to the microplate walls for cm^2^ of surface. Zosteric acid at sublethal concentrations induced a significant inhibition of cell adhesion compared to the control without the antifoulant. Adhered cells were reduced of ca. one order of magnitude by using 200 mg/L of the compound. The increase of the antifoulant concentration did not improve its performance.

### 2.5. Zosteric Acid Affected the Morphology, Thickness and Biomass of P. putida Biofilm

Visual inspection of *P. putida* biofilm grown in presence of zosteric acid highlighted different morphological development in comparison to the same cells without the antifoulant. *P. putida* biofilm without zosteric acid shows a flat development.

**Figure 4:**
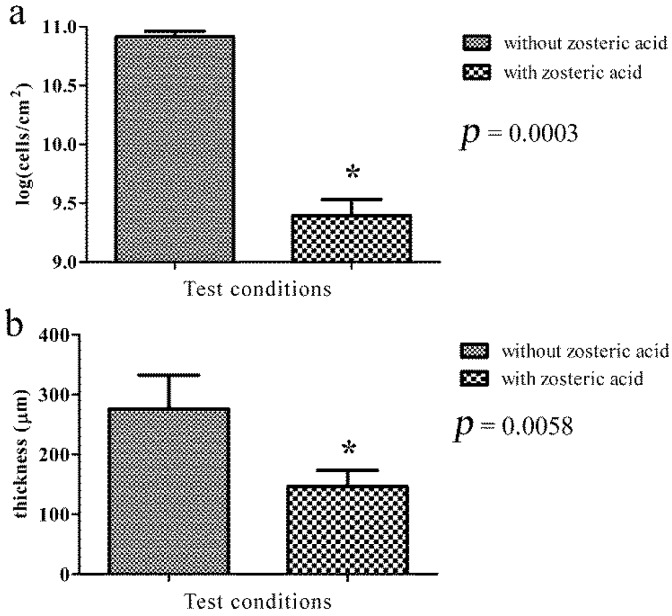
Effect of zosteric acid on the amount of *P. putida* sessile cells on polycarbonate membranes (**a**) and on the thickness of cryosectioned *P. putida* biofilm (**b**). Data represent the mean ± standard deviation of replicates; * statistically significant difference at the 95% confidence level between control and treated samples.

After 48 h of growth as a colony biofilm, a considerable decrease in biofilm formation was observed in the presence of the compound (200 mg/L). Treatment with zosteric acid produced biofilms containing 2.6 × 10^9^ ± 7.2 × 10^8^ CFU/cm^2^ (control 8.3 × 10^1^ ± 8.7 × 10^9^ CFU/cm^2^) (Figure 4a), leading to a cell reduction of 97%.

Measured biofilm thickness obtained by cryosectioning combined with microscopy analysis revealed that in the presence of zosteric acid, *P. putida* biofilm had a thickness of 146 ± 27 μm, significantly thinner than the film formed without it where the biofilm thickness was 276 ± 56 μm (Figure 4b). The yield reduction in biofilm thickness was by 50%.

Zosteric acid-treated biofilms retained similar morphological patterns to those observed in the control. The fluorescent staining procedure demonstrated the presence of the biofilm matrix and thus the formation of a biofilm. In both the control and the treated samples, Concanavalin A (ConA) staining resulted in an intense red fluorescent signal, revealing that the exopolysaccharide component of the matrix did not show quantitative changes in presence of zosteric acid.

### 2.6. General Features of the Zosteric Acid Behavior in the Environment

Different chemical and physical properties of zosteric acid were obtained with the goal of determining the general features of its behavior in the environment.

Zosteric acid is a practically odorless white crystalline powder that is readily soluble in water and has a high melting temperature (Table 3). Zosteric acid has low vapor pressure and octanol-water partition coefficient (Kow) close to zero that enhance its stability and presence in water.

**Table 3:** Physico-chemical properties of zosteric acid measured and/or predicted by EPIsuite (http://www.epa.gov/opptintr/exposure/pubs/episuite.htm).

Physico-chemical properties	Value	Source
Molecular weight (g/mol)	244	–
Melting point (°C)	260	[30]
Water solubility (mg/L at 20 °C)	2.5 × 10^5^	[21]
Vapor pressure (Pa, 25 °C)	3.81 × 10^−9^	Predicted
log Kow (octanol water coefficient, 20 °C)	−0.25	Measured in this work
log BCF (bioconcentration factor)	0.94	Predicted

### 2.7. Ecotoxicity Responses

The *D. magna* preliminary test showed no immobilization effects within the concentrations between 10 and 700 mg/L of zosteric acid. Taking into account this information, higher exposure concentrations were tested and in the final test no toxicity was observed even at 7000 mg/L zosteric acid. No significant variations of pH were observed during both exposures.

**Figure 5:**
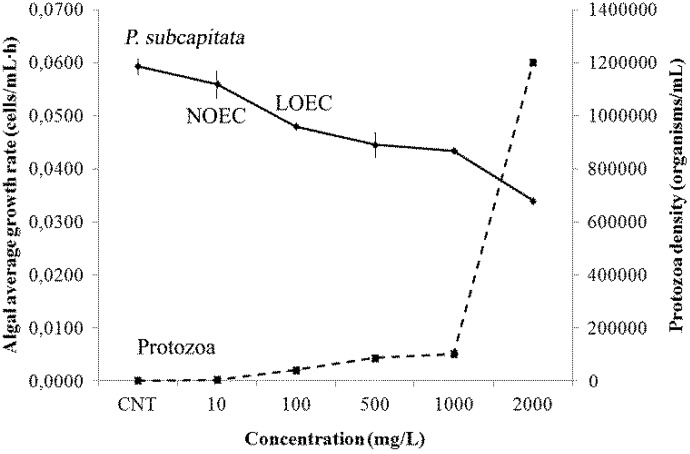
Algal average growth rate of *P. subcapitata* (cells/mL·h) and protozoa density (organisms/mL) at the different concentrations of zosteric acid and control. No observed effect concentration (NOEC) and low effect observed concentration (LOEC) on algal growth rate are also reported.

A significant effect on *P. subcapitata* algal growth was observed starting from 100 mg/L (ANOVA, *p* < 0.05) (Figure 5). A decrease of growth of algae was gradually observed with a simultaneous unexpected growing development of ciliates protozoa (with a density from 40,000 organisms/mL at 100 mg/L of zosteric acid to 1.2 × 10^6^ organisms/mL at the highest concentration).

## 3. Discussion

The present work was designed to provide a foundation for using zosteric acid as a strategy for mitigating biofilm formation on membranes in MBR of WWTPs equipped with membrane-based separation technology. It is worthwhile to keep in mind that the best way to apply this biocide-free anti-biofilm technology to preserve the structure and function of engineered systems would be to create a modified membrane by functionalizing its surface with the covalently bound non-toxic compound. The surface density of the compound depends on both its antibiofilm performance and its stability in the activated sludge of MBR plant. Villa *et al.* [20] have already demonstrated the efficiency of zosteric acid in hindering biofilm formation *in vitro* using different model microorganisms under different environmental conditions. Therefore, it was necessary to characterize the microbial communities in MBR in order to isolate and select the most suitable target microorganism as a model biofoulant to evaluate the potential of an antibiofilm strategy based on zosteric acid. This work represents the first step to transfer the antibiofilm performance of zosteric acid to the membrane based separation process technology in WWTPs.

Initial inspection by confocal laser scanning microscopy of fouled membranes from the Aldeno MBR plant highlighted the presence of sessile cells included in a matrix demonstrating that the biofouling was a considerable constituent of fouling process on such membranes as reported in several other works [31]. Observations demonstrated that microbial biofilms were constituted by separate agglomerates of cells with a good production of exopolysaccharides.

Bacterial communities from both sludge and fouled membranes were studied by culture independent methods in order to characterize bacterial taxa involved in the biofouling process. The sequences from sludge and fouled membranes were phylogenetically most closely related to alphaproteobacteria (64%), bacteroidetes (27%) and betaproteobacteria (9%), and to gammaproteobacteria (45%), betaproteobacteria (22%), acidobacteria (11%) and bacteroidetes (11%), respectively. In both cases, proteobacteria were found to be the most prevalent classes in agreement with several previous researches [32,33,34,35]. They are also known to play a major role in the development of membrane biofouling in WWTPs [36,37]. As PCA-analysis did not highlight significant similarities among bacterial communities (both in sludge and those that grew in sessile form on fouled membrane), it demonstrated that only some taxa present in sludge formed biofilm on the membranes and that the bacterial community causing biofouling can be different on the several point of membrane surface. Therefore, a bacterial collection of cultivable strains was obtained only from fouled membranes to investigate the antibiofilm activity of zosteric acid against fouler microorganisms. Many identified strains were previously reported in WWTPs. Members of the genus *Rhodococcus* have been isolated from a large range of habitats such as soil, rocks and ground water [38]. *Shewanella xiamenensis* was commonly isolated from fresh water and seawater [39,40]. *Bacillus arvi*, *B. cereus*, *Enterobacter cloacae*, *Lysinibacillus fusiformis*, and strains belongs to *Chitinimonas* and *Pseudomonas* genera were generally isolated from soil and/or rhizosphaera [41,42,43] and could have come from agricultural waste. Members of *Pseudomonas* genus were also frequently isolated from WWTPs [32,44], growing on several type of membranes [27].

The observed bacterial variety highlighted an extremely complex biofilm on fouled membranes. Therefore, for this work, a single representative strain was used as a target bacterium. These comprehensive phylogenetic analyses revealed that in this research Gammaproteobacteria resulted to be the prevalent taxon on fouled membrane both by culture dependent and independent methods (they represent 55% and 45% of identified bacterial community, respectively), in agreement with observations by Hörsch *et al*. by using fluorescence *in situ* hybridization [45]. The discrepancy in bacterial populations observed between this study and others, where alpha- and beta-proteobacteria resulted to be largest groups [32,34,35,36], could have been due to the differences in process configuration and experimental approaches. Gamma proteobacteria were considered to have a distinctive role in initial membrane colonization since they were more predominant than betaproteobacteria and alphaproteobacteria in primary biofilms as compared with mature ones [45]. In this case study, *Pseudomonas* was the prevalent genus belonging to gamma proteobacteria isolated from fouled membranes. *Pseudomonas* spp. are known for their relevant abundance in municipal wastewater and in activated sludge compared to other bacterial taxa, and for their ability to foul surfaces rapidly [46,47,48]. Therefore, *P. putida* (accession number HF546530) was chosen among the isolated *Pseudomonas* strains as target organism to study the antibiofilm properties of zosteric acid. Although Xu and coworkers [49] and Newby and coworkers [50] have already tested the effectiveness at preventing the attachment of *P. putida* planktonic cells, on the best of our knowledge this is the first time that the effects on the *P. putida* biofilm were investigated. In addition, for the first time the attachment experiments were performed simulating the chemical condition (chemical oxygen demand, COD; total phosphorus, *P*_tot_; and total nitrogen, *N*_tot_) measured in the wastewater of the MBR plant where the fouling strain was isolated.

Zosteric acid did not represent a carbon and energy source for *P. putida* at the sublethal concentrations tested and at 200 mg/L reduced cell adhesion by 97% on hydrophobic surfaces of 96-well plates without raising the selective pressure. Pitts *et al.* [51] claimed that a compound which could reduce biofilm by at least 40% can be considered as a satisfactory antibiofilm agent. Xu and colleagues [49] observed a reduction in bacterial biofilm coverage by 98.2% at 500 mg/L zosteric acid. The mathematical model developed by Villa and colleagues [20] predicted that 200 mg/L zosteric acid reduced cell adhesion on the hydrophobic surface by about 55% and 78%, for *E. coli* and *B. cereus* respectively, whereas 500 mg/L zosteric acid ensured a percentage reduction of bacterial adhesion of more than 90%. Taken together these results showed that, although zosteric acid exhibited a species-specific behavior, it always decreased bacterial adhesion, thus displaying potential as a broad range antifoulant.

For the first time, the membrane-supporting biofilm reactor was used to further investigate the effect of zosteric acid on *P. putida* biofilm formation. This technique permitted to force cells surface attachment, a feature that allowed to (i) mimic the membrane scenario in which foulers are retained by the membrane surface during gravity-driven filtration and (ii) directly investigate the effect of zosteric acid biofilm structural development and organization bypassing the effect on the adhesion phase. Although a biofilm-like structure was observed in presence of zosteric acid, *P. putida* biofilm development was impaired with significant decrease in biomass and mean thickness. These findings corroborated previous results that revealed the ability of zosteric acid to slow down surface colonization and reduce biofilm thickness [20,21].

Noteworthy, zosteric acid treatment induced a migration activity of the peritrichous flagellated bacterium *P. putida* over the polycarbonate surface not amenable to a biofilm phenotype. Recently, Villa *et al.* [20] reported the ability of zosteric acid treatment to induce a hyper-motile phenotype in *E. coli* by controlling the degree of flagellation in the swim cell-state. These results suggested that sub-lethal zosteric acid concentrations act as environmental cue driving the transition between motile and sessile lifestyle and making *E. coli* cells too motile for proper biofilm formation [20,52].

By identifying the surface colonizers and testing the efficacy of zosteric acid against the membrane-fouler *P. putida*, this study proposes a potentially interesting biocide-free approach for the prevention and reduction of biofilm formation in membrane-based separation processes. However, despite these promising properties to date no studies have addressed the fate and ecotoxicological effects of zosteric acidin the environment. To evaluate the suitability of zosteric acid in this study, for the first time, preliminary screening investigations and speculations about its potential environmental partitioning behavior and its ecotoxicity effects toward target aquatic organisms were performed. The basic physical and chemical properties that describe a chemical’s partitioning between solid, liquid and gas phases were estimated. These include melting point, vapor pressure, water solubility and the Kow. Considering the application of zosteric acid as antibiofilm agentin WWTP, the water ecosystem would be the principal recipient of primary emissions and fate processes. High water solubility and low Kow suggest that water likely retains in solution most of the compound introduced in the system and it is expected that this compound does not partition appreciably into soils, settling particles in water and sediments. It follows that any exposure via the sediments, while unlikely, would be due to zosteric acid dissolved in the pore water and the exposure of the terrestrial environment is to be expected being limited to areas subjected to flooding or intensive runoff and direct deposits of sewage or wastewater sludge. High water solubility and low vapor pressure suggest that volatilization of zosteric acid from surface water to the atmosphere is negligible. Finally, zosteric acid is fully biodegradable and possesses a half-life in seawater of a few days [53].

Because the Kow characterizes partitioning between aqueous and organic, lipid-like phases, it is used to estimate a variety of toxicological, and environmental fate parameters. One specific use of the Kow is as a gauge for the potential for bioaccumulation [54]. If a chemical tends to partition into the organic phase (is lipophilic), then the chemical can be stored in fatty tissue of fish and will bioaccumulate in animals that consume the fish [54]. The low Kow and the high water solubility of zosteric acid, suggests a low bioaccumulation potential. Predictions using the Arnot–Gobas bioconcentration factor (BCF) and bioaccumulation factor (BAF) model from the EPIsuite prediction software [55] clearly suggest that zosteric acid should not be expected to bioaccumulate either at the lower or upper trophic levels (BCF of 0.94) and it possesses the biotransformation half-life of 2 h. To date, only few toxicological data exist about the zosteric acid. Xu *et al.* [49] reported that zosteric acid EC50 values were 167 ± 3.9 and 375 ± 10 mg/L for *P. putida* and Lake Erie bacteria, respectively, and the EC50 value from the Microtox assay was 442 ± 100 mg/L. Using *in vitro* experiments with suitable primary cell based models, Villa *et al.* [21] demonstrated that 10 mg/L of zosteric acid did not compromise the cellular activity, adhesion, proliferation or morphology of either the murine fibroblast line L929 or the human osteosarcoma line MG-63. Flemming [53] reported no detectable LD_50_ toward larval fish, and an acute toxicity profile similar to table sugar. To our knowledge, until now, no data on the toxic effects of zosteric acid on freshwater invertebrates are available. Therefore, considering possible future uses and environmental fate of zosteric acid in freshwater environments, the importance of knowing the potential toxic effects on target aquatic organisms seems crucial. No toxicity effect toward *D. magna* at any tested concentration was observed. A significant inhibition of growth of algae was observed starting from 100 mg/L which represents the 72 h LOEC (Lowest Observed Effect Concentration); in our bioassay NOEC (No Observed Effect Concentration) was 10 mg/L. It has to be underlined that an increasing density of grazers protozoa was observed, probably contributing to the progressive decrease of algal density with increasing concentrations of zosteric acid. As no protozoa were observed in control flasks their presence may be connected to the exposure medium. Protozoa are well known to feed on algae [56,57,58] and their grazing effects on algal density cannot be excluded even in this case. As a consequence caution is requested in the evaluation of the toxic effects of zosteric acid on algal communities and in depth investigations should be performed to have more extended ecotoxicological data on algae and to evaluate if the growth inhibition is directly related to zosteric acid or to protozoa grazing activity. Taking into account that LOEC of algae is referred to a chronic exposure, these results can support the development of a biocide-free strategy based on zosteric acid to counteract unwanted biofilm in membrane-based separation processes.

## 4. Experimental Section

### 4.1. Sample Collection and Preparation

To characterize the fouling process, fouled filtration membranes were collected from the MBR WWTP of Aldeno (Trento, Italy), a plant fed with real municipal wastewater after sieving and degritting. Samples of about 1 cm^2^ of filtration membrane were taken from both fouled and apparently unfouled areas by sterile scalpel and transferred in sterile plates in laboratory. The samples of filtration membranes were suspended in tube with 900 μL of saline solution and vortexed for 5 min to remove sessile cells. Tubes were stored at −20 °C. Some filtration membrane samples both covered and uncovered of fouling were placed on glass slides, confined by *in situ* frames (1 cm^2^ area; Eppendorf, Hamburg, Germany) for microscopy analysis.

In addition, four samples of sludge (2 L each) were collected from the same MBR plant near to the panels with the filtration membranes and conserved in sterile bottles. Fifty milliliter of sludge samples were filtered by sterile 0.22 μm filters (Millipore, Milan, Italy). Filters were placed on sterile tubes with 1.8 mL of lysis buffer (EDTA 40 Mm, Tris–HCl 50 Mm pH 8, sucrose 0.75 M), vortexed for 5 min and stored at −20 °C for the DNA extraction.

The chemical-physical features of the influent wastewater during the sampling were as follows: temperature 13.1 °C, pH 7.7, COD 251 mg/L, biological oxygen demand (BOD) 55 mg/L, total suspended solids (TSS) 52 mg/L, *P*_tot_ 10.1 mg/L, *N*_tot_ 63.5 mg/L, ammonia nitrogen 35.6 mg/L, organic nitrogen 27.1 mg/L, nitric nitrogen 0.8 mg/L.

### 4.2. Analysis of Bacterial Community by Culture Independent Methods

Total DNA was extracted directly from microbial cells collected from sludge and from filtration membrane samples as described by Murray *et al.* [59] to characterize the fouling community.

Bacterial communities were analyzed amplifying 16S rRNA gene fragments with primers GC-357 F and 907 R [60] with the following chemical conditions: 1× of PCR Rxn buffer, 1.5 mM of MgCl_2_, 0.2 mM of dNTP mix, 0.3 μM of each primer and 1 U of Taq DNA polymerase (GoTaq, Promega, Milan, Italy). The thermal cycling program included an initial denaturation at 94 °C for 5 min followed by thirty-five cycles consisting of denaturation at 94 °C for 45 s, annealing at 56 °C for 45 s and extension at 72 °C for 2 min, and a final extension step at 72 °C for 10 min.

All PCR reactions were performed in a total volume of 50 μL containing 2 μL of template DNA. The amplicons obtained were separated by DGGE, as previously described by Polo *et al.* [60]. The electrophoresis run was performed for 15 h at 90 V by D-Code Universal Mutation Detection system (Bio-Rad, Milan, Italy). Amplicons from sludge and membrane samples were loaded in the same gel to make the DGGE profiles comparable. The DGGE was performed with the denaturant gradients 40%–60%. The gels were stained by SYBR-Green (Invitrogen, San Giuliano Milanese, Italy) and the results observed by Gel Doc (Bio-Rad, Milan, Italy) apparatus. The excised bands were eluted in 50 μL milli-Q water by incubation at 37 °C for 3 h and successively overnight at 4 °C. All the excised bands were identified by sequencing (Primm, Milan, Italy). The sequences were analyzed in June 2011 using BLASTN software [61] and deposited in the European Nucleotide Archive (ENA) [62] under the accession number HF546518 to HF546526 and HF546538 to HF546548.

Using 16S DGGE profiles, the line plots were generated by ImageJ software [63], and then imported into an Excel^®^ file as *x*/*y* values. The matrix were analyzed using PCA-analysis in order to study the relationship between bacterial communities in sludge and on filtration membranes. The multivariate investigations were conducted by XLSTAT (version 7.5.2, Addinsoft, Paris, France) on autoscaled data normalized using the Pearson correlation as similarity index. Line profile data were scaled in order to consider even the small peaks, downweighting the relative importance of the predominant bands [64]. The significance of the PCA-analysis model was tested by a cross-validation procedure.

### 4.3. Isolation and Identification of Bacterial Strains from Fouled Filtration Membrane

To isolate fouler strains, one 100 μL of cells suspensions collected from filtration membrane samples were inoculated in two cultural media: plate count agar medium (PCA; Merck, Dramstadt, Germany) for aerobic heterotrophic bacteria and on the agarized synthetic wastewater medium (SWA) reported by Khan *et al.* [65] modified in order to have the same COD, *N*_tot_ and *P*_tot_ values measured in the wastewater of the Aldeno MBR plant and added with agar (258.8 mg/L glucose, 242.6 mg/L NH_4_Cl, 43.9 mg/L KH_2_PO_4_, 10 mg/L CaCl_2_, 10 mg/L MgSO_4_·7H_2_O, 3 mg/L FeCl_3_, 2 mg/L MnCl_2_, 200 mg/L NaHCO_3_, agar 17 g/L, pH 7–8), with incubation at 30 °C for 3–5 days. All different colonies observed were isolated by subsequent plating. The genomic DNA of isolated strains was extracted as described by Murray *et al.* [59] and analyzed amplifying 16S rRNA gene fragments with primers 357 F and 907 R with the following chemical conditions: 1× of PCR Rxn buffer, 1.5 mM of MgCl_2_, 0.12 mM of dNTP mix, 0.3 μM of each primer and 1 U of Taq DNA polymerase (Invitrogen, San Giuliano Milanese, Italy). The thermal cycling program included an initial denaturation at 94 °C for 5 min followed by thirty-five cycles consisting of denaturation at 94 °C for 45 s, annealing at 56 °C for 45 s and extension at 72 °C for 2 min, and a final extension step at 72 °C for 10 min.

All PCR reactions were performed in a total volume of 50 μL containing 3 μL of template DNA. The sequences were analyzed in June 2011 using BLASTN software. The nucleotide sequences were deposited in the ENA under the accession number HF546527 to HF546537.

### 4.4. Synthesis of Zosteric Acid

Zosteric acid was prepared by treating a *N*,*N*-dimethylformamide solution of *trans*-4-hydroxycinnamic acid with the sulfur trioxide pyridine complex, as previously described by Villa *et al.* [20]. The obtained sodium zosterate contains <5% of sodium sulfate. Proton nuclear magnetic resonance spectroscopy of sodium zosterate showed a downfield shift of aromatic protons adjacent to the strong electron withdrawing sulphooxy group with respect to the same protons of *trans*-4-hydroxycinnamic acid [20].

### 4.5. Cellular Growth with Zosteric Acid

The ability of *P. putida* to grow on zosteric acid as sole carbon and energy source was tested using SWB medium without glucose and supplemented with zosteric acid at sublethal concentrations 200 and 600 mg/L (0.73 and 2.2 mM respectively), the same quantities used in later experiments [20]. The positive control was represented by the SWB supplemented with both 395.7 mg/L (2.2 mM) and 4000 mg/L (22.2 mM) of glucose [20]. Microbial growth was followed by determination of absorbance at a wavelength of 600 nm (OD_600_). All the experiments were repeated three times.

### 4.6. Microplate-Based Biofilm Assay

*P. putida* cells adhesion was assessed quantitatively using fluorochrome-labeled cells in hydrophobic 96-well blacksided plates as previously reported by Villa *et al.* [20]. Briefly, 200 μL of SWB containing 10^6^ cells and different sublethal concentrations (from 200 to 600 mg/L) of zosteric acid were placed in microtiter plate wells. After 72 h incubation at 30 °C, the microtiter plate wells were washed one times with 200 μL of phosphate buffer solution (PBS; Sigma-Aldrich, Milan, Italy) and adhered cells were firstly permeabilized with a 70% ethanol solution for 2 min and then stained using 10 μg/mL of DAPI (Sigma-Aldrich) in PBS for 30 min in the dark at room temperature. After three wash with 200 μL of PBS, fluorescence intensity was measured using the fluorometer VICTOR™ X Multilabel Plate Readers (PerkinElmer, Waltham, MA, USA) at excitation wavelength 433 nm and emission wavelength 335 nm. A standard curve of fluorescence intensity *vs.* cell number was determined and used to quantify the performance of the antibiofilm compound. For all the combinations a negative control without the potential antibiofilm agent was present.

Four measurements were performed for each combination and their average was computed.

### 4.7. Biofilm Preparation by Colony-Biofilm Method

*P. putida* biofilms were cultivated on sterile polycarbonate membrane filters (diameter, 2.5 cm; pore size, 0.2 μm; Whatman, Maidstone, UK) placed onto PCA plates using the colony biofilm protocol by Anderl *et al.* [66]. A tissue paper moistened with PBS or with PBS plus 200 mg/L zosteric acid was placed between the agar culture medium and the membrane filters for the control and the treated samples respectively and replaced every 24 h. After 48 h of incubation at 30 °C, the membrane-supported *P. putida* biofilms were visually inspected, photographed, removed, suspended in PBS and serially diluted in microtiter plates. Biofilm cells were enumerated using the drop-plate method in which 20 μL drops of each dilution were placed onto PCA plates. All the experiments were conducted in duplicate.

### 4.8. Biofilm Sectioning

The biofilms grown on polycarbonate membranes by colony-biofilm method were carefully covered with a layer of optimum cutting temperature (OCT formulation, Tissue-Tek^®^, Sakura, Alphen aan den Rijn, The Netherlands) and placed in dry ice until completely frozen. The frozen samples were dipped vertically into the center of a cryosectioning mold filled with fresh OCT. The frozen samples were sectioned at −19 °C using a CM1850 cryostat (Leica, Wetzlar, Germany). The 5-μm thick cryosections were mounted on glass slides treated by Vectabond (Vector laboratories, Peterborough, UK).

### 4.9. Biofilm Imaging by Confocal Laser Scanning Microscopy (CLSM)

Both biofilm on membrane filtration samples from the MBR WWTP and biofilm sections were stained with green-fluorescent nucleic acid stain SYTO9 (5 μM) (Invitrogen) and Texas red-labeled ConA (200 μg/mL; Invitrogen) for 10 min in the dark at room temperature. After incubation, samples from filtration membranes were rinsed three times with PBS. Samples were visualized using a Leica TCSNT CLSM with a 10× dry objective, excitation filters at 488 and 568 nm, and emission filters at 530 and 590 nm. The images were analyzed with the software Imaris (Bitplane Scientific Software, Zurich, Switzerland). All the experiments were conducted in duplicate. For the biofilm sections, the biofilm thickness of the control and treated samples was measured for each image at three different locations randomly selected along the profile. More than five images per sample were taken for microscope analysis. These measurements were used to calculate the average thickness and the associated standard deviation.

### 4.10. Physicochemical Profiling of Zosteric Acid

The chemical and physical properties of zosteric acid are reported in Table 3. The Kow of zosteric acid was determined by potentiometric titration with the Scirus GLpKa instrument coupled with a computer-aided system for the evaluation of acid dissociation constant (pK_a_) values (Scirus Refinement Pro software Ver. 1.0) as reported by Vistoli *et al.* [67]. To obtain the Kow, separate titrations of the compound were carried out using various volumes of *n*-octanol. In the presence of *n*-octanol, the pK value shifts as a consequence of the partitioning of the substance into the organic phase, allowing a new pK constant to be determined. These shifts in the pK values were used to determine log Kow, the logarithm of the partition coefficient of the neutral form [67].

### 4.11. D. magna Acute Ecotoxicity Test

Parthenogenetic *D. magna* were cultured (40 ind/L) in 1 L glass beaker filled with 500 mL of culture medium (hardness 240 mg/L, pH 7.8) [68], maintained at (20 ± 1) °C under 16-h light:8-h dark photoperiod. Culture medium was renewed and offspring discarded three times a week. In these occasions, daphnids were fed with a suspension of the unicellular green alga *P. subcapitata* (0- to 8-days-old daphnids: 8 × 10^6^ cells/(ind day) and from 8-days-old daphnids: 16 × 10^6^ cells/(ind day)) and the yeast *Saccharomyces cerevisiae* (15 × 10^6^ cells/(ind day)). Brood daphnids were renewed every 6 weeks and replaced with neonatal organisms. Daphnids from the third generation were used for culture renewal and for exposure experiments to reduce variability [68].

Stock solutions were prepared by dissolving the appropriate amount of zosteric acid in culture medium. Two acute toxicity tests were carried out. In a preliminary test, organisms were exposed to solutions within the range 10–700 mg/L, in the final test, zosteric acid concentrations were between 700 and 7000 mg/L in geometric progressions no higher than 2. Acute toxicity assays were performed following the OECD Guideline No. 202 [68]. Five newborn daphnids, less than 24 h of age, were exposed to 20 mL of test solution. Each exposure was run in four replicates for 48 h at (20 ± 1) °C without feeding. Daphnids immobilization was used as acute toxicity end-point after 24 and 48 h, when pH was also measured.

### 4.12. P. subcapitata Growth Inhibition Ecotoxicity Test

In order to adapt the test alga *P. subcapitata* to the test conditions, an inoculum culture in the test medium [69] was prepared 3 days before the start of the test. The algal density was adjusted in order to allow exponential growth to prevail in the inoculum culture until test start.

Test was carried out following the OECD guideline No. 201 [69]. Exponentially growing algal cultures were exposed to five concentrations ranging from 10 to 2000 mg/L of zosteric acid and control under standard conditions. Algal density of each exposure concentration was measured at 72 h by cell counting with a Burker counting chamber; the assay was run in three replicates starting from an algal density of 50,000 cells/mL. Exposure flasks containing 80 mL of test solution were maintained at (20 ± 1) °C under 16-h light:8-h dark photoperiod (7000 lux) with stirring to facilitate transfer of CO_2_ for 72 h. To be valid, test cells density in the control culture should have increased at least 16 times during the 72 h exposure period. Data are reported as the average specific growth rate [69] for each concentration, included control calculated as:

(1) $$\mu_{\text{i}-\text{j}}=\frac{\text{ln}\;X_{\text{j}}-\text{ln}\;X_{\text{i}}}{t_{\text{j}}-t_{\text{i}}}{\text{day}}^{-1}$$

where: *μ*_i–j_ is the average specific growth rate from time i to j, *X*_i_ is the density at time i, and *X*_j_ is the density at time j.

Protozoa density of each exposure concentration was measured at 72 h by organisms counting with a Burker counting chamber. A subsample of each flanks were fixed with methanol to immobilize organisms in order to facilitate the counting.

### 4.13. Statistical Analysis

For the microplate-based biofilm assay and toxicity data, the mean values, standard errors of the means and variance analysis with ANOVA of all replicates were calculated using GraphPad Prism 4 to assess the significance of differences in the results collected at several antifoulant concentration. Differences were considered significant with *p*-values <0.05. Individual comparisons were made post hoc with the Tukey–Kramer test.

For the biofilm assay, statistical significance of CFU/cm^2^ and biofilm thickness was determined by Student’s *t*-test analysis (*p* < 0.05).

## 5. Conclusions

In this study we characterized the biofilm microbial communities on the membranes of a submerged MBR plant, located in Aldeno, Trento, Italy and the membrane colonizer *P. putita* was selected as a biofilm target system to investigate the ability of zosteric acid in mitigating biofilm formation and the antifouling concentration to be used. On the best of our knowledge, this is the first time that the antibiofilm properties of zosteric acid were tested against an isolated bacterium from biologically fouled membranes of a submerged MBR plant at sublethal concentrations. These investigations exhibited that zosteric acid is effective in reducing biofilm formation by *P. putida* without raising the selective pressure. In addition, in view of an application, a preliminary screening of the potential environmental partitioning behavior of this promising molecule suggested that the water compartment represents the main environmental recipient and capacitor of this molecule while the fraction associated to any other compartment, including atmosphere, soils, settling particles in water and sediments would be virtually negligible. In addition, the low Kow and the high water solubility of zosteric acid suggested a low bioaccumulation potential. Finally, preliminary ecotoxicity tests suggested that zosteric acid does not represent a threat for the target aquatic organisms *D. magna* while for the impact on algal communities more ecotoxicological data are recommended.

This study encourages the use of zosteric acid-based methods as an innovative, effective, biocide-free and green strategy for the prevention and reduction of biofilm formation in membrane-based separation processes. However, the biodegradability of zosteric acid in freshwater, its potential toxicity toward a wider range of aquatic organisms, human and animals, and its consistent performance toward a bacterial consortium of sludge in a pilot scale MBR will have to be further investigated for optimization of its use on a field scale.
